# Association between loneliness and social isolation and health outcomes among cancer survivors and non-cancer controls

**DOI:** 10.3389/fragi.2026.1813024

**Published:** 2026-04-28

**Authors:** Nancy E. Avis, Sybil Crawford, Alicia Colvin

**Affiliations:** 1 Department of Social Sciences and Health Policy, Wake Forest University School of Medicine, Winston-Salem, NC, United States; 2 Tan Chingfen Graduate School of Nursing, UMass Chan Medical School, Worcester, MA, United States; 3 Department of Epidemiology, University of Pittsburgh, Pittsburgh, PA, United States

**Keywords:** anxiety, cancer, depressive symptoms, HRQL, loneliness, social isolation

## Abstract

**Introduction:**

The present analyses take advantage of an existing cohort of cancer survivors (cases) and women without a history of cancer (controls) to examine whether older cancer survivors experience greater loneliness or social isolation than women without a history of cancer and how loneliness and social isolation are related to health outcomes.

**Methods:**

Cross-sectional analyses were conducted from Visit 17 of The Study of Women’s Health Across the Nation (SWAN), a multiracial/ethnic cohort study initiated to study the biological and psychosocial changes occurring during the menopausal transition. We identified 276 women who had developed cancer over the 30 years of SWAN (eligible cases) and 1,123 women who never had cancer. Loneliness was measured using the UCLA 3-item loneliness scale. Social isolation was assessed with a modified version of the Social Network Index. Outcomes included health-related quality of life (HRQL) as assessed by the SF-36 (MCS and PCS), depressive symptoms (CES-D), and anxiety. Covariates included sociodemographic, health-related, and psychosocial variables. Associations of case-control status, years since diagnosis in cases, loneliness, and social isolation with MCS and PCS were estimated using analysis of covariance, and with depressive symptoms and high anxiety using logistic regression. Effect modification of loneliness and social isolation by case-control status was assessed by adding relevant interaction terms to models.

**Results:**

Case-control status was not significantly related to loneliness or social isolation. Loneliness and social isolation were negatively related to PCS before, but not after covariate adjustment. Corresponding negative associations with MCS remained statistically significant after covariate adjustment. Loneliness and social isolation were positively associated with depressive symptoms. Loneliness, but not social isolation, was positively associated with high anxiety. With the exception of a significantly stronger unadjusted association of PCS with loneliness in controls than in cases, there was no statistically significant effect modification by case-control status before or after covariate adjustment.

**Conclusion:**

Neither loneliness nor social isolation was related to cancer survivorship status. Although both loneliness and social isolation were related to HRQL and mental health outcomes in a cohort of older women, these associations were similar for cancer survivors and those without a history of cancer.

## 1 Introduction

Loneliness and social isolation are highly prevalent and increasingly recognized as important risks to healthy aging (e.g., Courtin and Knapp, 2017; Hawton et al., 2011; Perissinotto et al., 2012). Although prevalence estimates vary based on the population studied and the specific measures used, a 2018 population-based survey of U.S. adults aged 45 or older found that more than one-third were lonely based on the UCLA loneliness scale (Anderson and Thayer, 2018); the National Survey of Aging trends Study found 24% of older adults were considered socially isolated (Cudjoe et al., 2020). Although loneliness and social isolation are often treated synonymously, they are thought to represent separate domains of social functioning. Social isolation represents the structural domain (i.e., the amount of social contact and integration), while loneliness is the functional or subjective feeling (National Academies of Science and Medicine, 2020; Luo, 2023). Both loneliness and social isolation have been shown to be related to mental health and health-related quality of life (HRQL). Studies have shown that loneliness is related to depression (e.g., Akinyemi et al., 2025; Bruss et al., 2024; Courtin and Knapp, 2017; Luo, 2023), anxiety (Lim et al., 2016), and lower HRQL (Freak-Poli et al., 2022; Liu and Guo, 2007; Tan et al., 2020). Social isolation has also been related to depression (Luo, 2023; Santini et al., 2020), anxiety (Santini et al., 2020), and lower HRQL (Freak-Poli et al., 2022; Hawton et al., 2011) though the relationship to these outcomes has been shown to be stronger for loneliness than social isolation (Freak-Poli et al., 2022; Luo, 2023).

With growing interest in loneliness and social isolation, researchers have begun studying their impact on cancer patients and survivors. A systematic review and meta-analysis reported that 32%–47% of patients with cancer reported moderate levels of loneliness (Deckx et al., 2014) while the Health Information National Trends Survey (HINTS) estimated that 35.9% of adult cancer survivors experienced moderate to severe loneliness (Wheldon et al., 2025). Both loneliness and social isolation have been found to have detrimental effects on both physical and mental wellbeing of cancer patients and survivors. Similar to research in the general population, loneliness has been related to depression (Hyland et al., 2019; Kurisu et al., 2025; Liu et al., 2021; Pilleron et al., 2023), anxiety (Liu et al., 2021; Rentscher et al., 2021), and lower HRQL (Hyland et al., 2019) among cancer patients and survivors. Social isolation has been related to depression (Liu et al., 2021) and anxiety (Liu et al., 2021). Factors related to greater feelings of loneliness among adult cancer survivors include fair-poor overall health, depression, anxiety, and high psychological distress. Protective factors include being married and having greater social support (Deckx et al., 2014; Hyland et al., 2019; Pilleron et al., 2023; Wheldon et al., 2025). A systematic review of risk factors for loneliness in patients with cancer also found that longer time since diagnosis was positively related to loneliness (Deckx et al., 2014), which was also found in a study by Wheldon and colleagues (Wheldon et al., 2025), although other studies found no association (Wang et al., 2024). Other cancer-related factors such as cancer site, treatment type, or stage of cancer have not been associated with loneliness (Deckx et al., 2014).

Despite the large literature on cancer patients and survivors, the vast majority of this research does not include people without cancer. Only a handful of studies include non-cancer controls (Deckx et al., 2015; Friedman et al., 1989; Rentscher et al., 2021; White et al., 2024), though two of these studies were conducted in the midst of the COVID pandemic (Rentscher et al., 2021; White et al., 2024). Two studies found no difference in reported loneliness between cancer survivors and controls (Friedman et al., 1989; Rentscher et al., 2021) and one study did not find a difference after 6 months post treatment (Deckx et al., 2015). One study found that cancer survivors experienced decreased depression, anxiety, and loneliness during the COVID pandemic compared to those without a history of cancer (White et al., 2024), though this study focused on change scores over the course of the pandemic. Given that prevalence rates and many of the risk and protective factors are similar for the general population and for cancer survivors, it is important to determine if being a cancer survivor confers greater risk of loneliness and social isolation than for the general population.

The Study of Women’s Health Across the Nation (SWAN) provides the opportunity to address limitations of existing research. SWAN is a community-based cohort study of initially mid-aged women begun in 1994 with 17 follow-up visits. SWAN affords a cohort of women who developed cancer over the 30 years of follow-up along with comparator women who remained cancer free, and a large array of potential predictors. The present analyses take advantage of this existing cohort of women cancer survivors (cases) and women without a history of cancer (controls) to examine whether older cancer survivors experience greater loneliness and/or social isolation than women without a history of cancer and whether time since diagnosis is related to loneliness and social isolation among cancer survivors. We also examine the association between loneliness/social isolation and mental health outcomes including HRQL, depressive symptoms, and anxiety and whether these associations differ for cases and controls. We hypothesize the following: 1. Cancer survivors will not report greater loneliness and/or social isolation compared to women without a history of cancer, 2. loneliness and social isolation will be associated with lower HRQL and greater depressive symptoms and anxiety, and 3. the relationship between loneliness and social isolation and health outcomes will not differ between cancer survivors and controls.

## 2 Materials and methods

### 2.1 Study design

SWAN is a multiracial/ethnic cohort study characterizing biological and psychosocial changes occurring during the menopausal transition (Sowers et al., 2000). From 1995 to 97, seven clinical sites recruited non-Hispanic white women and women from one of four racial/ethnic minorities (Black, Japanese, Hispanic, or Chinese).

### 2.2 Study population

SWAN eligibility included age 42–52 years; an intact uterus and at least one ovary; not pregnant, lactating, using oral contraceptives, or hormone therapy; and having a menstrual cycle in the 3 months before screening (Sowers et al., 2000). Participants were assessed in-person at baseline and approximately annually through follow-up visit 17, using a standardized protocol that included medical, reproductive and menstrual history; lifestyle and psychosocial factors; physical and psychological symptoms; and anthropometric measurements. Instruments were translated into Spanish, Japanese, and Cantonese. Loneliness and social isolation were assessed at SWAN visit 17 conducted between 2021–2023.

SWAN *cases* are women who reported cancer other than non-melanoma skin cancer at the screening interview and/or at any annual follow-up visit. *Controls* had no cancer at baseline or during follow-up other than non-melanoma skin cancer. With the exception of breast and colon cancer, incident cancer status was obtained solely through participant self-report. Beginning at visit 12, we requested medical records from women reporting incident breast or colon cancer. Records were adjudicated for cancer diagnosis and treatment by a committee of physicians with expertise in women’s health and oncology. There was good agreement between self-reported breast/colon cancer and the adjudicated diagnosis among cases who had medical records available. Of the 157 cases for whom medical records were received, 150 (95.5%) were confirmed, 5 (3.2%) were indeterminate, and 2 (1.3%) were determined not to be cancer. If medical records were not available, the participant’s self-report of breast or colon cancer was used to determine incident cancer status for the current analyses.

When available, date of last cancer diagnosis was taken from medical records; otherwise, date of last cancer diagnosis was taken from annual interview self-report. Time between last cancer diagnosis and visit 17 was categorized as within 5 years, between 5 and 10 years, and more than 10 years.

Among 1626 SWAN participants at visit 17, 227 (14.0%) were omitted from analyses due to missing data on loneliness and/or social isolation (SI) (42 had no survey data, 174 completed an abbreviated survey that omitted questions on loneliness and SI, 11 completed the full survey but skipped loneliness and/or SI items). In the remaining 1,399 participants, we identified 276 (19.7%) cases (259 incident cancer, 17 cancer history reported at screening) and 1,123 (80.3%) women who never had cancer.

### 2.3 Measures

#### 2.3.1 Primary predictors

Loneliness was measured using the 3-item version of the University of California Los Angeles Scale (UCLA 3-item loneliness scale; Hughes et al., 2004), a well-validated and frequently used measure of loneliness (Dahlberg et al., 2022; Das et al., 2021). In this study, the Cronbach α of the UCLA loneliness scale was 0.89. Participants are asked the frequency of feeling lack of companionship, left out, and isolated from others on a 3-point scale from “hardly ever,” “some of the time,” to “often.” The total score ranges from 3 to 9 and higher scores indicate greater loneliness. Given that there is no consistently established cut-point for classifying people as lonely (Dahlberg et al., 2022) we chose to classify participants as lonely using the cut-point of 1.5 from the National Social Life, Health, and Aging Project (NSHAP) (Hawkley and Kocherginsky, 2018) where mean values greater than 1.5 represent loneliness. This corresponds to a frequency of “some of the time” for at least two items or “often” for at least one item.

Social isolation was assessed with a modified version of the Social Network Index (Cohen, 1991). The questionnaire assesses number and frequencies of 9 types of social contacts (children, parents/in-laws, other relatives, close friends, religious organization, employment, neighbors, volunteer work, other groups) in the past year. High contact roles are considered those where a person has contact with at least one person every 2 weeks. Total scores range from 0 to 9. We classified social integration on number of roles as low (0–3), moderate (4–5), and high (6–9) (Cohen et al., 1997) where the low category represents social isolation.

#### 2.3.2 Outcomes

Outcomes included HRQL, depressive symptoms, and anxiety. The SF-36 was used to assess HRQL with the original coding algorithm (raw scores transformed to a 0–100 range) (Ware et al., 1993). The SF-36 is a generic HRQL measure with 2 summary scores: the physical component summary (PCS) and the mental component summary (MCS) (Ware et al., 2000). The PCS and MCS are normalized so that a score of 50 represents the population average (Ware et al., 2000). In this study, Mosier’s reliability coefficient (Mosier, 1943; Widhiarso and Ravand, 2014) applicable to composite scores, was 0.93 for PCS and 0.86 for MCS.

We also examined presence versus absence of depressive symptoms (score ≥16 versus <16 on Center for Epidemiologic Studies Depression (CES-D) scale) (Radloff, 1977). In this study, Cronbach α of the continuous CES-D score was 0.86. Anxiety was based on the sum of 4 symptoms, including irritability, nervousness, heart racing, and fearfulness; in this study, the Cronbach α of the anxiety measure was 0.62. We dichotomized at 4+ versus <4, as a total score ≥4 indicates high anxiety (Bromberger et al., 2013).

#### 2.3.3 Covariates

Covariates included variables related to any of the four outcomes and/or to loneliness or social isolation in SWAN. All variables except educational attainment, race/ethnicity, study site, and trait anxiety were taken from V17. The set of covariates considered for each outcome were based on variables previously found to be related in SWAN (Avis et al., 2018; Bromberger et al., 2013; Kravitz et al., 2022; unpublished data from SWAN).

*Sociodemographic variables* included age, partner status (married or partnered versus non partnered), race/ethnicity, study site, educational attainment (≤high school, some college, ≥4 years college), and difficulty paying for basics (very or somewhat hard/not hard at all). Race/ethnicity was self-defined by respondents using an open-ended question: “How would you describe your *primary* racial or ethnic group?” Categories were non-Hispanic white, black, Chinese, Hispanic, or Japanese.

*Health-related* factors included number of chronic medical comorbidities other than cancer, self-assessed health, sleep problems (any of difficulty falling asleep, staying asleep, and/or early morning awakening ≥3 times/week in past 2 weeks, yes versus no), frequency of vasomotor symptoms (VMS) in past 2 weeks: none, 1–5 days, 6+ days (ordinal); frequency of vaginal dryness over the past 2 weeks (not at all, 1–5 days, 6–8 days, 9–13 days, or every day; ordinal), frequency of urinary incontinence, and psychotropic medications.

*Lifestyle and anthropometric variables* included current cigarette smoking (yes/no), body mass index (BMI) in continuous kg/m^2^, and non-occupational physical activity (Sternfeld et al., 1999). *Psychosocial* factors included perceived stress (Cohen et al., 1983), number of negative life events considered very upsetting from a list of 18 events (none, 1, ≥2) since last study visit, trait anxiety (Spielbergeret al., 1970), social support (Sherbourne and Stewart, 1991), positive and negative affect (PANAS), discrimination (Williams et al., 1997), history of childhood maltreatment (Childhood Trauma Questionnaire (CTG) (Bernstein et al., 1994), and death of close friend/relative.

### 2.4 Ethical considerations

All study sites obtained approval from their Institutional Review Boards. The study adhered to ethical guidelines, ensuring the confidentiality of participant data. All participants provided written informed consent.

### 2.5 Statistical analyses

Cases and controls were compared regarding outcomes and covariates using chi-square testing for categorical characteristics and two-sample t-tests for continuous characteristics. Associations of cancer status–ever/never cancer and years since last diagnosis in ever-cancer participants–with loneliness and SI were estimated using binomial and multinomial logistic regression, respectively. Depression and high anxiety also were modeled using binomial logistic regression. PCS and MCS were modeled using analysis of covariance (ANCOVA). Unadjusted associations were estimated, followed by covariate-adjusted associations. Effect modification by ever/never cancer was tested by including its interaction with loneliness and SI, respectively, in the models for depression, anxiety, and HRQL. Cell counts were too small to permit testing effect modification by years since diagnosis among cases.

## 3 Results

### 3.1 Sample characteristics of cancer survivors and controls

A total of 276 women were identified who ever had cancer. With few exceptions, cancer survivors were similar to controls on most variables. The exceptions were that cancer survivors had slightly lower general health and they were also more likely to report any urine leakage. Non-Hispanic white participants were overrepresented in the cancer group, relative to the controls (Table 1). The primary cancer site was breast (44.9%), with gynecologic cancer second (15.2%) (Supplementary Table S1).

**TABLE 1:** Characteristics of participants who ever versus never reported a cancer diagnosis.

Characteristic^a^	Ever cancer (N = 276)	Never cancer (N = 1,123)	p-value^b^
Race/ethnicity: % (N)	​	​	**0.0194**
Non-hispanic white	59.78 (165)	49.15 (552)	​
Non-hispanic black	20.29 (56)	24.93 (280)	​
Hispanic	5.07 (14)	4.63 (52)	​
Chinese	7.97 (22)	10.51 (118)	​
Japanese	6.88 (19)	10.77 (121)	​
Age: mean (SD)	72.31 (2.85)	72.00 (2.63)	0.0861
Baseline education, college or higher: % (N)	51.64 (142)	49.37 (550)	0.5012
Financial strain: % (N)	​	​	0.5784
Not at all hard	79.20 (217)	80.69 (894)	​
Somewhat or very hard	20.80 (57)	10.3 (214)	​
Self-reported health: % (N)	​	​	0.2507
Excellent/Very good	38.46 (105)	44.02 (493)	​
Good	41.39 (113)	37.59 (421)	​
Fair/Poor	20.15 (55)	18.39 (206)	​
Number of chronic conditions, excluding cancer: mean (SD)	3.08 (1.53)	2.98 (1.46)	0.3726
SF-36 PCS: mean (SD)	45.16 (10.54)	46.67 (10.24)	**0.0301**
SF-36 MCS: Mean (SD)	51.82 (9.83)	52.76 (9.20)	0.1382
Urinary incontinence, # days in past month: % (N)	​	​	0.0693
Never	20.80 (57)	26.74 (292)	​
Less than 1 day/week	34.31 (94)	36.45 (398)	​
Several days/week	24.45 (67)	19.60 (214)	​
Almost daily/daily	20.44 (56)	17.22 (188)	​
Any sleep-related problems: % (N)	43.48 (120)	41.39 (464)	0.5290
Current smoking: % (N)	3.64 (10)	4.20 (47)	0.6742
Physical activity: mean (SD)	7.30 (2.06)	7.42 (1.96)	0.3726
Social support: mean (SD)	13.58 (3.21)	13.42 (3.15)	0.4484
Currently married/partnered: % (N)	51.65 (141)	55.95 (625)	0.1998
Lonely: % (N)	30.07 (83)	32.50 (365)	0.4382
High social contacts: % (N)	​	​	0.5715
Low	20.65 (57)	17.99 (202)	​
Moderate	50.72 (140)	53.25 (598)	​
High	28.62 (79)	28.76 (323)	​
CES-D depression: % (N)	12.00 (33)	11.14 (124)	0.6872
High anxiety: % (N)	14.13 (39)	12.65 (141)	0.5106
Perceived stress: mean (SD)	6.43 (2.51)	6.56 (2.46)	0.4454
# very stressful life events: % (N)	​	​	0.4924
0	60.36 (166)	58.43 (655)	​
1	17.09 (47)	20.25 (227)	​
2+	22.55 (62)	21.32 (239)	​

^a^ Concurrent unless otherwise specified.

^b^ 2-sample t-test for continuous variables, chi-square test for categorical variables.

p-values <.05 are bolded.

There was no difference between cancer survivors and controls in the percentage of women who were classified as lonely or socially isolated (Table 1). Among cancer survivors, 30.1% were considered lonely and 20.7% socially isolated. Percentages among controls were 32.5% and 18% respectively.

### 3.2 Relationship between cancer status and outcomes

Tables 2, 3 show the relationship between cancer status and loneliness (Table 2) and social isolation (Table 3) unadjusted and covariate-adjusted. Ever-diagnosis was unrelated to loneliness, before and after covariate adjustment; In the ever-cancer group, loneliness was significantly less prevalent within 5 years of last diagnosis (22.3%) compared with >10 years beyond last diagnosis (36.7%), with an intermediate prevalence of 32.7% for 5–10 years beyond last diagnosis. This pairwise difference remained statistically significant after covariate adjustment (18.5% versus 32.3%, p = 0.048), although the overall p-value was >0.05 both before and after covariate adjustment (0.06 and 0.14, respectively). Cancer diagnosis (ever/never) and years since last diagnosis were unrelated to social isolation, with relative risk ratios close to 1, before and after covariate adjustment.

**TABLE 2:** Associations of cancer and years since last diagnosis with loneliness: results from binomial logistic regression.

Cancer status and years since diagnosis	Unadjusted		Covariate-adjusted^a^	
	Odds ratio (95% CI)	p-value	Odds ratio (95% CI)	p-value
Ever cancer	​	0.4384	​	0.5182
Yes	0.89 (0.67, 1.19)	​	0.89 (0.63, 1.26)	​
No	Reference	​	Reference	​
Years since last cancer diagnosis^b^	​	0.0617	​	0.1422
<5 years	0.50 (0.27, 0.90)	​	0.48 (0.23, 0.99)	​
5–10 years	0.84 (0.42, 1.66)	​	0.73 (0.31, 1.74)	​
>10 years	Reference	​	Reference	​

^a^ Adjusted for: race/ethnicity, site, social support, married/partnered, discrimination, perceived stress, CES-D, 3-category life events.

^b^ Participants ever-diagnosed with cancer only.

**TABLE 3:** Associations of cancer and years since last diagnosis with social isolation: results from multinomial logistic regression.

Cancer status and years since diagnosis	Unadjusted			Covariate-adjusted^a^		
	Relative risk ratio (95% CI), low vs. High contacts	Relative risk ratio (95% CI), moderate vs. High contacts	Overall p-value	Relative risk ratio (95% CI), low vs. High contacts	Relative risk ratio (95% CI), moderate vs. High contacts	Overall p-value
Ever cancer	​	​	0.5720	​	​	0.9077
Yes	1.15 (0.79, 1.69)	0.96 (0.70, 1.30)	​	1.02 (0.67, 1.54)	0.95 (0.66, 1.37)	​
No	Reference	Reference	​	Reference	Reference	​
Years since last cancer diagnosis^b^	​	​	0.6010	​	​	0.5641
<5 years	0.80 (0.37, 1.74)	1.39 (0.75, 2.57)	​	0.99 (0.37, 2.61)	1.59 (0.79, 3.21)	​
5–10 years	0.95 (0.38, 2.36)	1.11 (0.52, 2.38)	​	0.95 (0.29, 3.15)	1.11 (0.48, 2.58)	​
>10 years	Reference	Reference	​	Reference	Reference	​

^a^ Adjusted for: race/ethnicity, site, age, college education (yes/no), physical activity, CES-D, death of close friend/relative.

^b^ Participants ever-diagnosed with cancer only.

### 3.3 Relationship between loneliness and social isolation and outcomes

Tables 4, 5 show the mean scores of the PCS and MCS according to being lonely/not lonely and level of social isolation (Table 4) and the percentage of women classified as having depressive symptoms and anxiety (Table 5) by loneliness and social isolation. Prior to covariate adjustment, lonely participants had significantly lower PCS and significantly lower MCS than non-lonely participants, with mean differences of −3.91 and −8.64 points, respectively. Covariate adjustment attenuated these differences (−0.70 and −3.73, respectively) and the difference in PCS was no longer statistically significant. Greater social isolation was significantly associated with lower PCS prior to covariate adjustment (mean difference between low and high of −2.68), but not after covariate adjustment (corresponding adjusted mean difference of −0.25). Social isolation was significantly associated with lower MCS (mean difference between low and high of −4.49) and although covariate adjustment attenuated the between-group differences (corresponding adjusted mean difference of −2.81), the overall p-value remained at <0.0001.

**TABLE 4:** Associations of loneliness and social isolation with HRQL: results from analysis of covariance.

Loneliness and social isolation status	Mean (Standard error)			
	PCS		MCS	
	Unadjusted	Covariate-adjusted^a^	Unadjusted	Covariate-adjusted^b^
Lonely				
Yes	43.71 (0.52)	46.63 (0.47)	46.70 (0.49)	50.14 (0.37)
No	47.62 (0.32)	47.33 (0.31)	55.34 (0.24)	53.87 (0.24)
p-value	<0.0001	0.2269	<0.0001	<0.0001
Social isolation = # of high contacts				
Low	44.77 (0.64)	46.76 (0.60)	49.68 (0.57)	50.91 (0.47)
Moderate	46.34 (0.38)	47.29 (0.34)	52.72 (0.34)	52.71 (0.27)
High	47.45 (0.51)	47.01 (0.45)	54.17 (0.46)	53.72 (0.37)
p-value	0.0049	0.7097	<0.0001	<0.0001

^a^ Adjusted for: race/ethnicity, site, financial strain, current smoking, comorbidity score (continuous), trouble sleeping, 3-category vaginal dryness, urinary incontinence frequency, physical activity, BMI, PANAS positive, PANAS negative, age.

^b^ Adjusted for: race/ethnicity, site, self-reported health, trouble sleeping, upsetting life events, perceived stress, trait anxiety, age.

**TABLE 5:** Associations of loneliness and social isolation with depressive symptoms and anxiety: results from binomial logistic regression.

Loneliness and social isolation status	Odds ratio (95% CI)			
	Depressive symptoms		Anxiety	
	Unadjusted	Covariate-adjusted^a^	Unadjusted	Covariate-adjusted^b^
Lonely				
Yes	7.60 (5.22, 11.06)	4.11 (2.55, 6.63)	3.77 (2.73, 5.21)	2.75 (1.93, 3.93)
No	Reference	Reference	Reference	Reference
p-value	<0.0001	<0.0001	<0.0001	<0.0001
Social isolation = # of high contacts				
Low	3.13 (1.93, 5.06)	2.20 (1.18, 4.10)	1.16 (0.75, 1.80)	0.91 (0.56, 1.48)
Moderate	1.40 (0.90, 2.17)	1.15 (0.66, 2.00)	0.83 (0.57, 1.19)	0.72 (0.48, 1.08)
High	Reference	Reference	Reference	Reference
p-value	<0.0001	0.0183	0.2292	0.2434

^a^ Adjusted for: race/ethnicity, site, age, anxiety, social support, trouble sleeping, SF-36, role physical, vasomotor symptoms, history of childhood maltreatment.

^b^ Adjusted for: race/ethnicity, site, age, psychotropic medications, self-reported health, comorbidities (continuous), stressful life events, financial strain, vasomotor symptoms.

Loneliness was strongly and statistically significantly positively associated with both depressive symptoms and anxiety, before and after covariate adjustment (Table 5). Unadjusted prevalences of depressive symptoms (lonely versus not lonely) were 26.1% versus 4.4% and of anxiety were 24.0% versus 7.7%. Corresponding adjusted prevalences were attenuated but still large, at 13.2% versus 3.6% and 17.0% versus 6.9%, respectively. Social isolation was also associated with depressive symptoms, with unadjusted prevalences ranging from 7.5% for high contacts to 20.3% for low contacts. Corresponding covariate-adjusted between-group differences were smaller (5.1% for high contacts versus 10.5% for low contacts) but remained statistically significant (overall p-value = 0.02). In contrast, social isolation was not related to anxiety, with unadjusted prevalences ranging from 11.6% for moderate contacts to 15.6% for low contacts, and covariate-adjusted prevalences ranging from 8.9% for moderate contacts to 11.9% for high contacts.

### 3.4 Effect modification by cancer status

**FIGURE 1:**
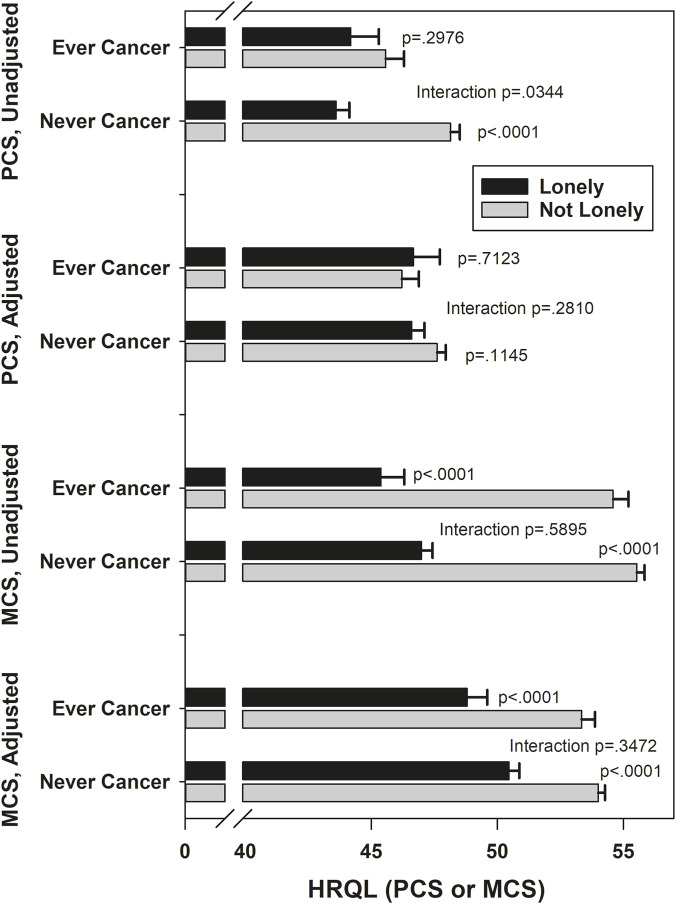
Mean HRQL by loneliness (lonely versus not lonely), by ever/never cancer. Adjusted PCS means adjusted for race/ethnicity, site, financial strain, current smoking, number of comorbidities, trouble sleeping, vaginal dryness, urinary incontinence frequency, physical activity, body mass index, PANAS positive, PANAS negative, age. Adjusted MCS means adjusted for race/ethnicity, site, self-reported health, trouble sleeping, stressful life events, perceived stress, trait anxiety, age.

**FIGURE 2:**
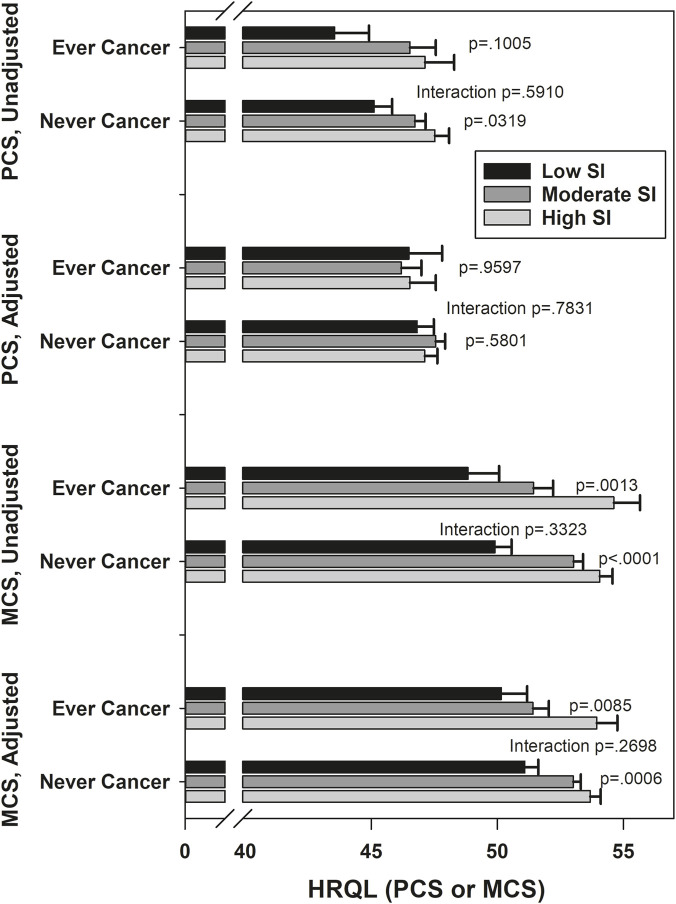
Mean HRQL by social integration (low, moderate, high), by ever/never cancer. Adjusted PCS means adjusted for race/ethnicity, site, financial strain, current smoking, number of comorbidities, trouble sleeping, vaginal dryness, urinary incontinence frequency, physical activity, body mass index, PANAS positive, PANAS negative, age. Adjusted MCS means adjusted for race/ethnicity, site, self-reported health, trouble sleeping, stressful life events, perceived stress, trait anxiety, age.

**FIGURE 3:**
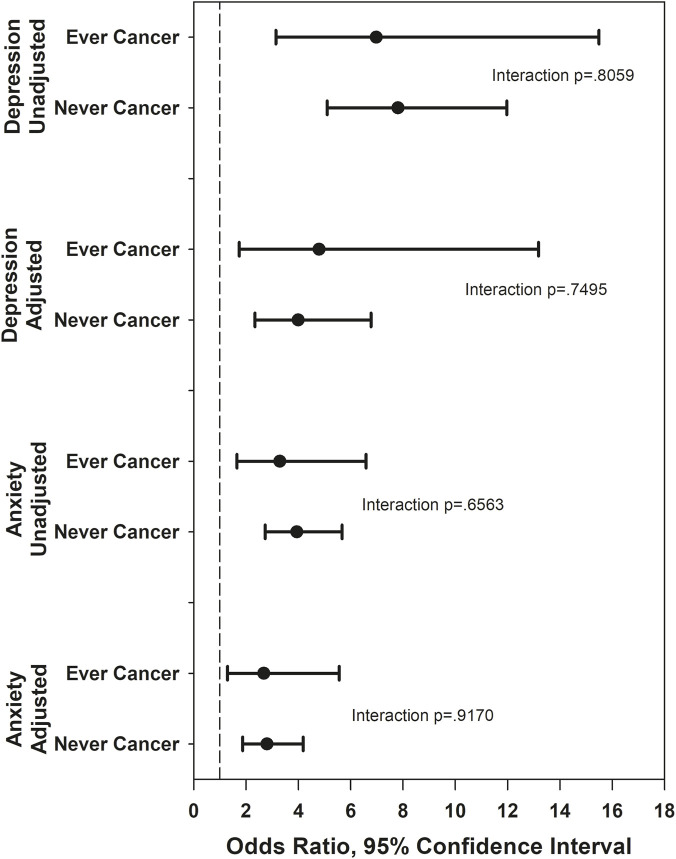
Odds ratios for lonely versus not lonely, depression and anxiety. Adjusted odds ratios for depression adjust for race/ethnicity, site, anxiety, social support, trouble sleeping, SF-36 role physical, vasomotor symptoms, history of childhood maltreatment, age. Adjusted odds ratios for anxiety adjust for race/ethnicity, site, psychotropic medications, self-reported health, number of comorbidities, stressful life events, financial strain, vasomotor symptoms, age.

**FIGURE 4:**
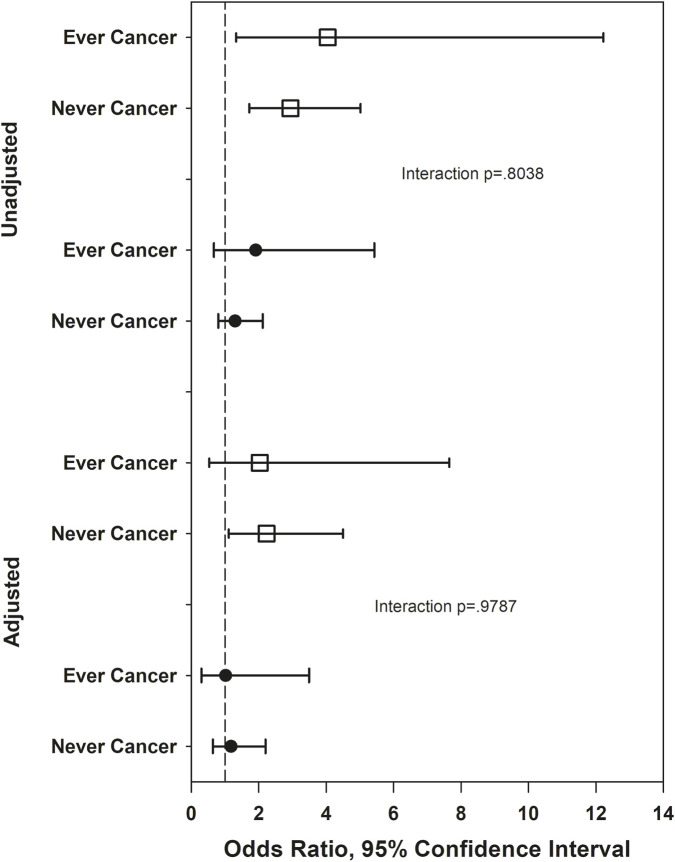
Odds ratios for low social integration (open squares) and moderate social integration (solid circles) versus high social integration, depression outcome. Adjusted odds ratios adjust for race/ethnicity, site, anxiety, social support, trouble sleeping, SF-36 role physical, vasomotor symOdds ratios for low social integration (open squares) and moderate social integration (solid circles) versus high social integration, depression outcome. Adjusted odds ratios adjust for race/ethnicity, site, anxiety, social support, trouble sleeping, SF-36 role physical, vasomotor symptoms, history of childhood maltreatment, age. ptoms, history of childhood maltreatment, age.

**FIGURE 5:**
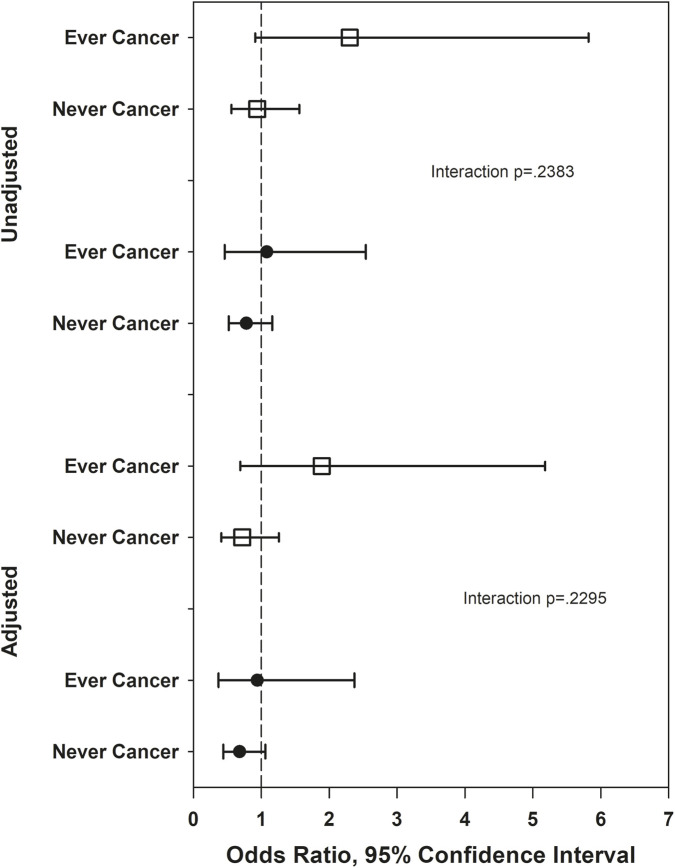
Odds ratios for low social integration (open squares) and moderate social integration (solid circles) versus high social integration, anxiety outcome. Adjusted odds ratios adjust for race/ethnicity, site, psychotropic medications, self-reported health, number of comorbidities, stressful life events, financial strain, vasomotor symptoms, age.

Figures 1–5 show the results of effect modification of loneliness and social isolation by cancer status for HRQL (Figures 1, 2) and depressive symptoms and anxiety (Figures 3–5).

Before covariate adjustment, the lonely vs. not lonely difference in PCS was statistically significantly larger in those never diagnosed with cancer (mean difference 4.54) than in those ever diagnosed (mean difference 1.39, interaction p = 0.03), but the interaction was smaller (mean differences −0.46 and 1.01, respectively) and no longer statistically significant after covariate adjustment (p = 0.28). With the above exception, there was no statistically significant effect modification by ever/never diagnosed of the associations of loneliness and social isolation with HRQL, with magnitudes of effect modification (differences of differences) between 0.67 and 1.63. There was no statistically significant effect modification by ever/never cancer of the associations of loneliness and social isolation with depression and anxiety, either before or after covariates. For depressive symptoms, the absolute magnitudes of effect modification were all under 3% for both loneliness and social isolation, before and after covariate adjustment. For anxiety, the absolute magnitude of effect modification for loneliness was under 3% before and after covariate adjustment. The absolute magnitude of effect modification for social isolation (low versus high contacts) was larger, at 12.2% unadjusted and 10.3% adjusted, although neither effect modification was statistically significant.

## 4 Discussion

In this study comparing older women cancer survivors with women without a history of cancer, there was no statistically significant difference between these two groups in the percentage classified as lonely or socially isolated in either unadjusted or adjusted analyses. Among cancer survivors, 30.1% were considered lonely and 20.7% socially isolated. Percentages among controls were 32.5% and 18% respectively. It is difficult to make prevalence comparisons to other studies, given differences in how loneliness and social isolation are measured and sample characteristics, but the percentage of women considered lonely is very similar to the 30% reported by Hawkley and Kocherginsky (2018) with the same 3-item UCLA measure and cut-point in their study of older adults. Among cancer survivors, those who were more than 10 years post diagnosis were more likely to be lonely compared to survivors <5 years after diagnosis even after adjustment. Wheldon and colleagues found that those diagnosed >5 years ago had increased odds of moderate to severe loneliness and suggested that this may be due to a reduction in social support further from diagnosis (Wheldon et al., 2025), although we found no significant decline in social support by time since diagnosis.

Consistent with other studies we found that both loneliness and social isolation were related to the MCS and depressive symptoms in both unadjusted and adjusted analyses (Hyland et al., 2019; Kurisu et al., 2025; Liu et al., 2021; Pilleron et al., 2023). Although both loneliness and social isolation were related to the PCS in unadjusted results, neither was significant when covariate-adjusted. Additional analyses of the covariates suggested that adjusting for positive affect and physical activity were likely the primary covariates accounting for the attenuation of the PCS-loneliness association. Other studies of the PCS that found loneliness related to lower scores on the PCS even after covariate adjustment adjusted for fewer variables than in the present study and did not include positive affect (Freak-Poli et al., 2022; Tan et al., 2020). In all studies (including the present analyses), the magnitude of the relationship to loneliness was greater for the MCS than the PCS. Although loneliness was related to anxiety in both unadjusted and adjusted results, social isolation was not related to anxiety in either unadjusted or adjusted analyses, contrary to other studies (Liu et al., 2021; Rentscher et al., 2021). It is noteworthy that one study was conducted among female inpatients diagnosed with breast cancer and the prevalence of anxiety was quite high (70.44%) (Liu et al., 2021) and the other was conducted during the COVID-19 pandemic (Rentscher et al., 2021) when people were generally isolated. In both of these studies, however, there was no difference between cancer survivors and controls.

Importantly, we found no statistically significant effect modification by ever/never cancer of the associations of loneliness and social isolation with the MCS, depression, and anxiety, either before or after covariate adjustment and only for unadjusted PCS. This suggests that neither loneliness nor social isolation convey greater risk for lower HRQL or greater depression or anxiety for older cancer survivors than for women without cancer.

This research has several limitations. It is possible that we failed to find effect modification by cancer status due to low power. There were fewer than 300 ever-cancer participants and they were divided into subgroups for loneliness and social isolation. However, inspection of the odds ratios suggests that associations tended to be similar in magnitude for ever- and never-cancer participants, in terms of both direction and magnitude of odds ratios, i.e., the p-values for interactions appear to be reflecting lack of effect modification and not simply small cell counts. Importantly, we acknowledge that these cross-sectional analyses do not address the temporal relationships between loneliness and social isolation and depression, anxiety, and HRQL and these relationships are likely bidirectional. Finally, except for cancers of the breast and colon, cancer diagnosis was based on self-report and not adjudicated.

Despite these limitations, the present research has a number of strengths. We were able to compare cancer survivors to women without a history of cancer from the same cohort of older women, used validated measures of both loneliness and social isolation, assessed several outcomes (HRQL, depressive symptoms, anxiety), and adjusted for a range of covariates. Our results demonstrate that both loneliness and social isolation are related to mental health outcomes in older women, but these relationships do not differ for cancer survivors compared to women without a history of cancer. We also found that the relationships between loneliness and social isolation and our outcomes are stronger for loneliness than social isolation.

## Data Availability

The datasets presented in this article are not readily available because in a first step, no data will be made available to researchers external to SWAN to allow primary researchers to fully exploit the dataset. The data will be shared in a second step according to a controlled access system. Requests to access the datasets should be directed to https://agingresearchbiobank.nia.nih.gov/.

## References

[B1] Akinyemi O. Abdulrazaq W. Fasokun M. Ogunyankin F. Ikugbayigbe S. Nwosu U. (2025). The impact of loneliness on depression, mental health, and physical well-being. PLoS One 20 (7), e0319311. 10.1371/journal.pone.0319311 40632698 PMC12240311

[B2] Anderson G. O. Thayer C. E. (2018). Loneliness and social connections: a national survey of adults 45 and older. Washington, DC: AARP Research. 10.26419/res.00246.001

[B3] Avis N. E. Colvin A. Bromberger J. T. Hess R. (2018). Midlife predictors of health-related quality of life in older women. J. Gerontol. A Biol. Sci. Med. Sci. 73 (11), 1574–1580. 10.1093/gerona/gly062 29596565 PMC6175022

[B4] Bernstein D. P. Fink L. Handelsman L. Foote J. Lovejoy M. Wenzel K. (1994). Initial reliability and validity of a new retrospective measure of child abuse and neglect. Am. J. Psychiatry 151 (8), 1132–1136. 10.1176/ajp.151.8.1132 8037246

[B5] Bromberger J. T. Kravitz H. M. Chang Y. Randolph J. F. Jr. Avis N. E. Gold E. B. (2013). Does risk for anxiety increase during the menopausal transition? Study of Women's health across the nation. Menopause 20, 488–495. 10.1097/GME.0b013e3182730599 23615639 PMC3641149

[B6] Bruss K. V. Seth P. Zhao G. (2024). Loneliness, lack of social and emotional support, and mental health issues - united States, 2022. MMWR Morb. Mortal. Wkly. Rep. 73 (24), 539–545. 10.15585/mmwr.mm7324a1 38900690 PMC11199020

[B7] Cohen S. (1991). “Social supports and physical health,” in Life-span developmental psychology: perspectives on stress and coping. Editors Greene A. L. Cummings N. Karraker K. H. (Hillsdale, NJ: Erlbaum Associates).

[B8] Cohen S. Kamarck T. Mermelstein R. (1983). A global measure of perceived stress. J. Health Soc. Behav. 24 (4), 385–396. 6668417

[B9] Cohen S. Doyle W. J. Skoner D. P. Rabin B. S. Gwaltney J. M. Jr . (1997). Social ties and susceptibility to the common cold. J. Am. Med. Assoc. 277 (24), 1940–1944. 9200634

[B10] Courtin E. Knapp M. (2017). Social isolation, loneliness and health in old age: a scoping review. Health Soc. Care Community 25 (3), 799–812. 10.1111/hsc.12311 26712585

[B11] Cudjoe T. K. M. Roth D. L. Szanton S. L. Wolff J. L. Boyd C. M. Thorpe R. J. (2020). The epidemiology of social isolation: national health and aging trends study. J. Gerontol. B Psychol. Sci. Soc. Sci. 75 (1), 107–113. 10.1093/geronb/gby037 29590462 PMC7179802

[B12] Dahlberg L. McKee K. J. Frank A. Naseer M. (2022). A systematic review of longitudinal risk factors for loneliness in older adults. Aging Ment. Health 26 (2), 225–249. 10.1080/13607863.2021.1876638 33563024

[B13] Das A. Padala K. P. Crawford C. G. Teo A. Mendez D. M. Phillips O. A. (2021). A systematic review of loneliness and social isolation scales used in epidemics and pandemics. Psychiatry Res. 306, 114217. 10.1016/j.psychres.2021.114217 34644661 PMC8502233

[B14] Deckx L. van den Akker M. Buntinx F. (2014). Risk factors for loneliness in patients with cancer: a systematic literature review and meta-analysis. Eur. J. Oncol. Nurs. 18 (5), 466–477. 10.1016/j.ejon.2014.05.002 24993076

[B15] Deckx L. van den Akker M. van Driel M. Bulens P. van Abbema D. Tjan-Heijnen V. (2015). Loneliness in patients with cancer: the first year after cancer diagnosis. Psychooncology 24 (11), 1521–1528. 10.1002/pon.3818 25914244

[B16] Freak-Poli R. Ryan J. Tran T. Owen A. McHugh Power J. Berk M. (2022). Social isolation, social support and loneliness as independent concepts, and their relationship with health-related quality of life among older women. Aging Ment. Health 26 (7), 1335–1344. 10.1080/13607863.2021.1940097 34219569

[B17] Friedman G. Florian V. Zernitsky-Shurka E. (1989). The experience of loneliness among young adult cancer patients. J. Psychosoc. Oncology 7 (3), 1–15. 10.1300/j077v07n03_01

[B18] Hawkley L. C. Kocherginsky M. (2018). Transitions in loneliness among older adults: a 5-year follow-up in the national social life, health, and aging project. Res. Aging 40 (4), 365–387. 10.1177/0164027517698965 29519211 PMC6355458

[B19] Hawton A. Green C. Dickens A. P. Richards S. H. Taylor R. S. Edwards R. (2011). The impact of social isolation on the health status and health-related quality of life of older people. Qual. Life Res. 20 (1), 57–67. 10.1007/s11136-010-9717-2 20658322

[B20] Hughes M. E. Waite L. J. Hawkley L. C. Cacioppo J. T. (2004). A short scale for measuring loneliness in large surveys: results from two population-based studies. Res. Aging 26 (6), 655–672. 10.1177/0164027504268574 18504506 PMC2394670

[B21] Hyland K. A. Small B. J. Gray J. E. Chiappori A. Creelan B. C. Tanvetyanon T. (2019). Loneliness as a mediator of the relationship of social cognitive variables with depressive symptoms and quality of life in lung cancer patients beginning treatment. Psychooncology 28 (6), 1234–1242. 10.1002/pon.5072 30932275

[B22] Kravitz H. M. Colvin A. B. Avis N. E. Joffe H. Chen Y. Bromberger J. T. (2022). Risk of high depressive symptoms after the final menstrual period: the study of Women's health across the nation (SWAN). Menopause 29 (7), 805–815. 10.1097/GME.0000000000001988 35796553 PMC9268212

[B23] Kurisu K. Okamura M. Ozawa K. Harashima S. Yoshiuchi K. Uchitomi Y. (2025). Impact of loneliness on depression among cancer survivors: a comparison between adolescents and young adults and other age groups. BMC Cancer 25 (1), 1319. 10.1186/s12885-025-14734-4 40818941 PMC12357444

[B24] Lim M. H. Rodebaugh T. L. Zyphur M. J. Gleeson J. F. (2016). Loneliness over time: the crucial role of social anxiety. J. Abnorm. Psychol. 125 (5), 620–630. 10.1037/abn0000162 27124713

[B25] Liu L. J. Guo Q. (2007). Loneliness and health-related quality of life for the empty nest elderly in the rural area of a mountainous county in China. Qual. Life Res. 16 (8), 1275–1280. 10.1007/s11136-007-9250-0 17703375

[B26] Liu B. Wu X. Shi L. Li H. Wu D. Lai X. (2021). Correlations of social isolation and anxiety and depression symptoms among patients with breast cancer of Heilongjiang province in China: the mediating role of social support. Nurs. Open 8 (4), 1981–1989. 10.1002/nop2.876 33939294 PMC8186692

[B27] Luo M. (2023). Social isolation, loneliness, and depressive symptoms: a twelve-year population study of temporal dynamics. J. Gerontology Ser. B, Psychol. Sci. Soc. Sci. 78 (2), 280–290. 10.1093/geronb/gbac174 36315577 PMC9938924

[B28] Mosier C. (1943). On the reliability of a weighted composite. Psychometrika 8 (3), 161–168. 10.1007/bf02288700

[B29] National Academies of Sciences Engineering and Medicine (2020). Social isolation and loneliness in older adults: opportunities for the health care system. Washington, D.C.: National Academies Press. 32510896

[B30] Perissinotto C. M. Stijacic Cenzer I. Covinsky K. E. (2012). Loneliness in older persons: a predictor of functional decline and death. Arch. Intern Med. 172 (14), 1078–1083. 10.1001/archinternmed.2012.1993 22710744 PMC4383762

[B31] Pilleron S. Sun V. Ayala A. P. Haase K. R. Arthur E. K. Kenis C. (2023). Loneliness in older adults living with cancer: a scoping review of the quantitative and qualitative evidence on behalf of the international society of geriatric oncology nursing and allied health interest group. J. Geriatr. Oncol. 14 (5), 101519. 10.1016/j.jgo.2023.101519 37179207 PMC10641442

[B32] Radloff L. S. (1977). The CES-D scale: a self-report depression scale for research in the general population. Appl. Psychol. Meas. 1 (3), 385–401. 10.1177/014662167700100306

[B33] Rentscher K. E. Zhou X. Small B. J. Cohen H. J. Dilawari A. A. Patel S. K. (2021). Loneliness and mental health during the COVID-19 pandemic in older breast cancer survivors and noncancer controls. Cancer 127 (19), 3671–3679. 10.1002/cncr.33687 34161601 PMC8419003

[B34] Santini Z. I. Jose P. E. Cornwall E. Y. Koyanagi A. Nielsen L. Hinrichsen C. (2020). Social disconnectedness, perceived isolation, and symptoms of depression and anxiety among older Americans (NSHAP): a longitudinal mediation analysis. Lancet Public Health 5 (1), e62–e70. 10.1016/S2468-2667(19)30230-0 31910981

[B35] Sherbourne C. D. Stewart A. L. (1991). The MOS social support survey. Soc. Sci. Med. 32 (6), 705–714. 10.1016/0277-9536(91)90150-b 2035047

[B36] Sowers M. F. Crawford S. Sternfeld B. Morganstein D. Gold E. B. Greendale G. (2000). “Design, survey sampling and recruitment methods of SWAN: a multi-center, multi-ethnic, community-based cohort study of women and the menopausal transition,” in Menopause: biology and pathobiology. Editors Lobo R. Marcus R. (New York: Academic Press), 175–188.

[B37] Spielberger C. D. Gorsuch R. L. Lushene R. E. (1970). STAI manual for the stait-trait anxiety inventory (“self-evaluation questionnaire”). Palo Alto, California: Consulting Psychologists Press.

[B38] Sternfeld B. Ainsworth B. E. Quesenberry C. P. (1999). Physical activity patterns in a diverse population of women. Prev. Med. 28 (3), 313–323. 10.1006/pmed.1998.0470 10072751

[B39] Tan S. S. Fierloos I. N. Zhang X. Koppelaar E. Alhambra-Borras T. Rentoumis T. (2020). The association between loneliness and health related quality of life (HR-QoL) among community-dwelling older citizens. Int. J. Environ. Res. Public Health 17 (2), 600. 10.3390/ijerph17020600 31963427 PMC7013468

[B40] Wang C. Qiu X. Yang X. Mao J. Li Q. (2024). Factors influencing social isolation among cancer patients: a systematic review. Healthcare 12 (10), 1042. 10.3390/healthcare12101042 38786452 PMC11120751

[B41] Ware J. E. Snow K. K. Kosinski M. Gandek B. (1993). SF-36 health survey: manual and interpretation guide. Boston: Health Institute, New England Medical Center.

[B42] Ware J. E. Kosinski M. Dewey J. E. Gandek B. (2000). SF-36 health survey: manual and interpretation guide. Lincoln RI: Quality Metric Inc.

[B43] Wheldon C. W. Shahsavar Y. Choudhury A. McCormick B. P. Albertorio-Díaz J. R. (2025). Loneliness among adult cancer survivors in the United States: prevalence and correlates. Sci. Rep. 15 (1), 3914. 10.1038/s41598-025-85126-8 39890855 PMC11785765

[B44] White L. L. Goldberg S. R. Spencer Feigelson H. Burnett-Hartman A. N. (2024). Depression, anxiety, and loneliness among cancer survivors during the COVID-19 pandemic. J. Psychosoc. Oncol. 42 (2), 242–255. 10.1080/07347332.2023.2238192 37486169

[B45] Widhiarso W. Ravand H. (2014). Estimating reliability coefficient for multidimensional measures: a pedagogical illustration. Rev. Psychol. 21 (2), 111–121.

[B46] Williams D. R. Yan Y. Jackson J. S. Anderson N. B. (1997). Racial differences in physical and mental health: socio-economic status, stress and discrimination. J. Health Psychol. 2, 335–351. 10.1177/135910539700200305 22013026

