# Supplementary material for: Association between loneliness and social isolation and health outcomes among cancer survivors and non-cancer controls
Source: Front Aging. 2026 Apr 28;7:1813024. doi: 10.3389/fragi.2026.1813024 (PMC13160904; doi:10.3389/fragi.2026.1813024)
Supplement: Supplementary file 1 [file Table1.docx]

**Supplemental Table 1: Characteristics of Participants Who Ever versus**

**Never Reported a Cancer Diagnosis**

| **Characteristic^(a)^** | **Ever Cancer (N=276)** | **Never Cancer (N=1123)** | **p-value^(b)^** |
| --- | --- | --- | --- |
| *Sociodemographics* |  |  |  |
| Race/ethnicity: % (N) |  |  | **.0194** |
| Non-Hispanic White | 59.78 (165) | 49.15 (552) |  |
| Non-Hispanic Black | 20.29 (56) | 24.93 (280) |  |
| Hispanic | 5.07 (14) | 4.63 (52) |  |
| Chinese | 7.97 (22) | 10.51 (118) |  |
| Japanese | 6.88 (19) | 10.77 (121) |  |
| Age: mean (SD) | 72.31 (2.85) | 72.00 (2.63) | .0861 |
| Baseline education, college or higher: % (N) | 51.64 (142) | 49.37 (550) | .5012 |
| *Missing* | 1 | 9 |  |
| Financial strain: % (N) |  |  | .5784 |
| Not at all hard | 79.20 (217) | 80.69 (894) |  |
| Somewhat or very hard | 20.80 (57) | 10.3 (214) |  |
| *Missing* | 2 | 15 |  |
|  |  |  |  |
| *Health-related* |  |  |  |
| Self-reported health: % (N) |  |  | .2507 |
| Excellent / Very good | 38.46 (105) | 44.02 (493) |  |
| Good | 41.39 (113) | 37.59 (421) |  |
| Fair / Poor | 20.15 (55) | 18.39 (206) |  |
| *Missing* | *3* | *3* |  |
| Number of chronic conditions, excluding cancer: mean (SD) | 3.08 (1.53) | 2.98 (1.46) | .3726 |
| *Missing* | 3 | 9 |  |
| SF-36 PCS: mean (SD) | 45.16 (10.54) | 46.67 (10.24) | **.0301** |
| *Missing* | 3 | 2 |  |
| SF-36 MCS: Mean (SD) | 51.82 (9.83) | 52.76 (9.20) | **.1382** |
| *Missing* | 3 | 2 |  |
| SF-36 role physical: mean (SD) | 65.22 (39.71) | 69.42 (39.67) | .1152 |
| Urinary incontinence, # days in past month: % (N) |  |  | .0693 |
| Never | 20.80 (57) | 26.74 (292) |  |
| Less than 1 day / week | 34.31 (94) | 36.45 (398) |  |
| Several days / week | 24.45 (67) | 19.60 (214) |  |
| Almost daily / daily | 20.44 (56) | 17.22 (188) |  |
| *Missing* | 2 | 31 |  |
| Vasomotor symptoms, # days in past 2 weeks: % (N) |  |  | .8025 |
| None | 73.72 (202) | 73.52 (819) |  |
| 1 – 5 days | 20.07 (55) | 19.21 (214) |  |
| 6+ days | 6.20 (17) | 7.27 (81) |  |
| *Missing* | 2 | 9 |  |
| Vaginal dryness, # days in past 2 weeks: % (N) |  |  | .1602 |
| None | 71.38 (197) | 74.08 (829) |  |
| 1 – 5 days | 9.42 (26) | 11.17 (125) |  |
| 6+ days | 19.20 (53) | 14.75 (165) |  |
| *Missing* | 0 | 4 |  |
| Any sleep-related problems: % (N) | 43.48 (120) | 41.39 (464) | .5290 |
| *Missing* | 0 | 2 |  |
| Current smoking: % (N) | 3.64 (10) | 4.20 (47) | .6742 |
| *Missing* | 1 | 3 |  |
| Physical activity: mean (SD) | 7.30 (2.06) | 7.42 (1.96) | .3726 |
| Missing | 7 | 29 |  |
| Years since last cancer diagnosis: % (N) |  |  | -- |
| Within 5 years | 40.58 (112) | -- |  |
| More than 5, less than or equal to 10 | 19.93 (55) | -- |  |
| More than 10 | 39.49 (109) | -- |  |
| Never diagnosed | -- | 1123 (100.0) |  |
| Site of last cancer diagnosis: % (N) |  | -- | -- |
| Breast | 44.93 (124) |  |  |
| Melanoma | 17.39 (48) |  |  |
| Gynecologic | 15.22 (42) |  |  |
| Colorectal | 5.07 (14) |  |  |
| Blood | 3.26 (9) |  |  |
| Renal | 2.90 (8) |  |  |
| Lung | 2.17 (6) |  |  |
| Thyroid | 1.45 (4) |  |  |
| Liver | 1.09 (3) |  |  |
| Salivary | 0.72 (2) |  |  |
| Stomach | 0.72 (2) |  |  |
| Throat | 0.36 (1) |  |  |
| Bladder | 0.36 (1) |  |  |
| Pancreas | 0.36 (1) |  |  |
| Eye | 0.36 (1) |  |  |
| Bone | 0.36 (1) |  |  |
| Other, non-specified | 2.90 (8) |  |  |
| Missing^(c)^ | 0.36 (1) |  |  |
|  |  |  |  |
| *Social* |  |  |  |
| Social support: mean (SD) | 13.58 (3.21) | 13.42 (3.15) | .4484 |
| *Missing* | 1 | 10 |  |
| Currently married / partnered: % (N) | 51.65 (141) | 55.95 (625) | .1998 |
| *Missing* | 3 | 6 |  |
| Lonely: % (N) | 30.07 (83) | 32.50 (365) | .4382 |
| High social contacts: % (N) |  |  | .5715 |
| Low | 20.65 (57) | 17.99 (202) |  |
| Moderate | 50.72 (140) | 53.25 (598) |  |
| High | 28.62 (79) | 28.76 (323) |  |
|  |  |  |  |
| *Psychological* |  |  |  |
| CES-D depression: % (N) | 12.00 (33) | 11.14 (124) | .6872 |
| *Missing* | 1 | 10 |  |
| High anxiety: % (N) | 14.13 (39) | 12.65 (141) | .5106 |
| *Missing* | 0 | 8 |  |
| STAI Trait Anxiety: Mean (SD) | 16.61 (4.84) | 16.38 (4.67) | .4756 |
| *Missing* | 8 | 37 |  |
| Perceived stress: mean (SD) | 6.43 (2.51) | 6.56 (2.46) | .4454 |
| *Missing* | 1 | 12 |  |
| # very stressful life events: % (N) |  |  | .4924 |
| 0 | 60.36 (166) | 58.43 (655) |  |
| 1 | 17.09 (47) | 20.25 (227) |  |
| 2+ | 22.55 (62) | 21.32 (239) |  |
| *Missing* | 1 | 2 |  |
| Childhood abuse / trauma: % (N) | 47.49 (104) | 51.33 (482) | .3057 |
| *Missing* | 57 | 184 |  |

^(a)^Concurrent unless otherwise specified

^(b)^2-sample t-test for continuous variables, chi-square test for categorical variables

^(c)^Reported only at cohort screening interview, site not available
